# Methodical Review of the Psychometric Properties of the Soft Skills Questionnaire for Nurses

**DOI:** 10.3390/healthcare14070827

**Published:** 2026-03-24

**Authors:** Joana Gutiérrez García, Silvia Ortíz Molina, Ricardo Pocinho, Juan José Fernández Muñoz

**Affiliations:** 1 Doctoral International School, Rey Juan Carlos University, 28935 Móstoles, Spain; j.gutierrezg.2023@alumnos.urjc.es; 2 University Hospital of Móstoles, 28935 Mostoles, Spain; silvia.ortiz@salud.madrid.org; 3 Polytechnic Institute of Leiria, High School of Education and Social Sciences, 2400 Leiria, Portugal; ricardo.pocinho@ipleiria.pt; 4 Area of Methodology of Behavioral Sciences, Universidad Rey Juan Carlos, 28922 Alcorcón, Spain

**Keywords:** soft skills, validation, communication skills, confidence, emotional intelligence

## Abstract

**Aims:** To conduct an exploratory analysis the psychometric properties of the Spanish version of the Soft Skills Questionnaire for Nurses (SSQN) and examine its conceptual coherence and its preliminary empirical behavior among nursing professionals and students. The aim is to critically assess the instrument’s suitability as a tool for exploring perceptions and self-reported soft skills rather than to establish its psychometric validity. **Design:** Exploratory methodological study focused on analyzing the empirical performance and conceptual adequacy of the SSQN within a Spanish sample, with particular attention to the internal patterns of responses and the coherence between the instrument’s items and its proposed dimensions. **Methods:** The process included the translation of the questionnaire and an empirical application in a sample of nursing professionals and students. Exploratory analyses were performed, including exploratory factor analysis and reliability assessment (Cronbach’s alpha and McDonald’s omega), using Jeffreys’s Amazing Statistics Program (JASP) (version 1.18.3), in order to examine the structural performance of the instrument and detect possible conceptual and methodological limitations. **Results:** The SSQN showed notable inconsistencies in its empirical structure, with dimensions that did not display clear or theoretically coherent patterns. Factor inconsistencies and low internal consistency suggest that the instrument does not adequately capture the multidimensionality of interpersonal skills, reflecting weaknesses inherent in its original formulation rather than in the adaptation process. **Conclusions:** The Spanish version of the SSQN cannot be considered valid or reliable in its current form. The results underscore the need for a thorough revision of the questionnaire and a conceptual rethinking to develop more robust tools for assessing soft skills. **Impact:** This study highlights the need for a solid methodological evaluation before introducing instruments designed to measure complex and subjective competencies in the healthcare field.

## 1. Introduction

In professional nursing practice, mastery of the so-called soft skills has become established as an essential component of holistic care. In a healthcare setting marked by increasing technological advancements, care delivery pressure, and clinical complexity, competencies such as effective communication, empathy, emotional self-regulation, critical thinking, and teamwork are fundamental to ensuring safe, ethical, and person-centered care [1,2]. These skills not only complement clinical knowledge but also define the quality of therapeutic relationships and the ability to adapt in the face of adverse situations.

Although the SSQN [3] was originally developed and validated in English, there is no version available for use in Spanish-speaking contexts. Having a Spanish translation would allow its applicability in educational and clinical settings to be explored, as well as assessing whether its conceptual structure is adequate for describing the competencies it aims to measure. However, before considering its implementation in these contexts, it is necessary to conduct a preliminary examination of its conceptual and empirical behavior, as certain elements of the original development indicate possible areas for improvement that could influence its use in other cultural and linguistic contexts.

Despite the growing recognition of the importance of such skills, the objective and rigorous assessment of soft skills in care settings continues to be a methodological challenge. Their cross-cutting, subjective, and highly contextualized nature makes it difficult to develop valid and reliable instruments, which limits their systematic incorporation into selection, training, and professional-development processes [4]. In response to this difficulty, other fields such as business have developed strategies such as situational judgment tests (SJTs) [5], competency-based structured interviews, 360° evaluations, or direct observation of performance, which could provide useful frameworks for adapting to the healthcare environment [6].

The SSQN, developed by Aridi et al. (2023) [3], has emerged as a promising tool for assessing these competencies in nursing. The questionnaire integrates threekey dimensions: interpersonal communication, emotional management, decision-making, and teamwork. Nevertheless, despite the relevance of its approach and the initial efforts to develop a specific instrument, its original validation process shows limitations that justify critical analysis, particularly regarding its internal consistency, structural coherence, and intercultural suitability.

Soft skills constitute a broad, multidimensional construct that encompasses interpersonal, emotional, ethical, and cognitive competencies required for effective professional performance. Although literature often includes domains such as communication, teamwork, emotional regulation, decision-making, and professional behavior, there is no universally accepted taxonomy, and the boundaries between dimensions frequently overlap. Within this conceptual diversity, the SSQN operationalizes the construct through three subscales: communication, management and emotional intelligence, and confidentiality. While this structure does not capture the full spectrum of soft skills described in the theoretical literature, it reflects those domains considered most essential in clinical training and professional–patient interaction. In particular, the inclusion of “confidentiality” aligns with the ethical and professional components of soft skills, understood as competencies related to responsibility, integrity, and appropriate handling of sensitive information—elements increasingly recognized as core components of professional soft-skill profiles. For the purposes of this study, soft skills are therefore defined as a set of interpersonal, emotional, and ethical competencies that support safe, effective, and patient-centred care, and the three-dimensional structure of the SSQN is interpreted as a pragmatic operationalization of this construct.

Given conceptual diversity, this study had a dual purpose. First, to offer a cultural and linguistic translation of the SSQN for possible use in Spanish-speaking educational and clinical contexts. Second, this study explores its conceptual alignment and empirical behavior, examining whether its three-dimensional structure reflects the competencies described in the literature. Rather than conducting a validation study, this research adopted a critical perspective to explore the consistency between the theoretical foundations of soft skills and the empirical structure proposed by the original instrument. Consequently, the study analyzed whether the three-dimensional model of the SSQN meaningfully represents the competencies associated with soft skills in nursing, using a sample of nursing professionals and students.

## 2. Method

### 2.1. Study Design

A methodological study was conducted in three phases: cultural and linguistic translation, pilot testing, and critical examination of the instrument’s psychometric properties.

### 2.2. Participans

Sample 1 was composed of 252 professional nurses from a University Hospital; 12.7% were male and 87.3% were female. The average age was 41.47 (SD = 10.27), with an age range of 22–65. Regarding professional categories, 90.87% were general nurses and the rest of the participants belonged to other areas (pediatric, community, or mental health). Most participants (36.05%) reported working morning/night shifts, followed by 22.61% on afternoon/night shifts, and 18.25% with fixed morning shifts. Rotating shifts were reported at 14.28%, while 5.10% worked only afternoon shifts and 3.17% had 12 h shifts. Most respondents (78.57%) held full-time positions, whereas 21.43% had reduced working hours. In terms of professional experience, 15.87% had less than 10 years, 20.23% had between 10 and 20 years, and 48.01% had over 20 years of experience. Regarding employment status, 60.71% were permanent staff, 12.69% were interim, and 26.58% held temporary contracts

Sample 2 consisted of 118 undergraduate nursing students enrolled at a Spanish university. The distribution by year was: 14.70% in the first year, 28.0% in the second year, 22.90% in the third year, and 34.40% in the fourth year. Although all participants belonged to different nursing schools, they had all been interns at the Mostoles University Hospital. Participation was voluntary and open to students from different academic years.

The inclusion criteria were: (a) being enrolled in the nursing degree program at the time of data collection; (b) being 18 years of age or older; and (c) providing informed consent after receiving information about the study objectives. Exclusion criteria included: (a) not wishing to participate and (b) not attending practical classes on the day of data collection.

The sample had a mean age of 22.18 years (SD = 4.22), ranging from 19 to 43 years. Of the participants, 11.86% were male and 88.14% were female. In addition, 67.97% had not received training in palliative care, while 32.20% had completed a course related to this area.

### 2.3. Procedure

Firstly, a direct translation procedure was established using an initial translator and a second translator who compared the results for conceptual equivalence. After that, an independent researcher reviewed the consistency, accuracy, and clarity of the translated items.

A direct translation procedure was conducted following international recommendations for instrument adaptation. First, two independent translators produced parallel forward translations: one native speaker of the target language and one professional familiar with the conceptual content of the questionnaire. Both versions were compared and synthesized into a single consensual version, ensuring conceptual equivalence between the original and translated items [7]. Both translations were then synthesized into a single consensual version by the research team. Subsequently, an independent researcher reviewed the preliminary version to assess the consistency, accuracy, and clarity of the translated items.

The difference in sample size between nursing professionals and students is explained by the organizational structure of the hospital. Each floor of the center corresponds to a specific service, and only a limited proportion of students rotate through these services during their clinical placements. This distribution naturally results in a smaller sample size for the student group, whose presence is temporary and designed to allow closer supervision and a more individualized learning process. Therefore, the asymmetry between both groups does not reflect a methodological bias but rather, structural and educational characteristics inherent to the functioning of the hospital.

Secondly, a pilot study was conducted with the participation of the Nursing Department and supervisors from different areas of the hospital, with 24 participants. Each participant was contacted individually via corporate email and received a detailed explanation on how to complete the questionnaire. Preliminary analyses were carried out to assess the performance of the items in the scales included in the protocol. In this sense, it was observed that several items presented response difficulties, which did not align with the findings of previous studies.

Thirdly, the questionnaire was distributed via the Google Forms platform starting in December 2024 to all nursing staff at the hospital collaborating on the research project. The sampling procedure was non-probabilistic and incidental. Additionally, several researchers conducted in-person visits to the center to increase the number of nursing professionals. The response rate was 96%. The same protocol was also sent to a sample of students from a Spanish university who voluntarily chose to participate in the research project. All data from both samples were treated anonymously and in accordance with confidentiality principles.

Finally, the research project was approved by the Research Ethics Committee of the University Hospital of Mostoles on 26 September 2024, under registration number CEI2024/039. All participants signed an informed consent form to access the questionnaire.

### 2.4. Intruments

Sociodemographic variables, for instance, gender, age, marital status, and professional categories for both samples. These variables were developed ad hoc for this psychometric study.

Soft Skills Questionnaire [3]. This scale was designed to evaluate various aspects related to interaction and personal and professional performance in the healthcare setting. The questionnaire consists of 25 items with a five-point Likert scale format, where 1 = strongly disagree and 5 = strongly agree.

The instrument is structured in three dimensions: Communication skills (13 items), which measure effectiveness, clarity, and reciprocity in information exchange processes. The original instrument reported Cronbach’s alpha of 0.867 for this dimension. The second dimension is management level and emotional intelligence (6 items). This dimension explores skills related to organization, decision-making, and emotional regulation. It allows for the assessment of self-awareness, emotional control, empathy, and competencies associated with managing tasks or specific situations. The Cronbach’s alpha of the original scale was 0.621. The third dimension is confidentiality (6 items). This dimension focuses on assessing responsibility, ethics, and commitment in handling sensitive information, as well as respect for privacy in various contexts. The internal consistency index of the original scale was α = 0.730

Maslach Burnout Inventory Human Services Survey (MBI-HSS) [8] is a standardized tool developed to assess burnout in professionals working in human services, such as healthcare or education. It consists of 22 items divided into three key dimensions: emotional exhaustion (Cronbach’s alpha = 0.89), depersonalization (Cronbach’s alpha = 0.59), and personal accomplishment (Cronbach’s alpha = 0.74). The answer scale is a seven-point Likert scale where participants are asked how often they experience symptoms, with 1 = never and 7 = every day. Data on test–retest reliability for the three-dimensions ranges between 0.53 and 0.82. This instrument has been validated in many countries and organizational contexts [9,10,11,12,13,14,15].

### 2.5. Analysis

Statistical procedures were performed using JASP 0.18.3 [16]. For the exploratory factor analysis (EFA) of each dimension [3], several assumptions were checked beforehand, including the Kaiser–Meyer–Olkin (KMO) index, Bartlett’s test of sphericity [17], large sample size, multivariate normality, linearity, and inter-item correlations [18]. These steps ensure that the data are suitable for factor extraction and that the resulting structure is robust.

The maximum likelihood method was used for factor extraction, combined with an Oblimin oblique rotation, as factors in psychological and health contexts are often correlated. To determine the optimal number of factors, Horn’s parallel analysis was applied [19], which is considered more reliable than traditional methods such as Kaiser’s criterion.

The internal consistency of the total scale and its dimensions were assessed using Cronbach’s alpha, McDonald’s omega, and item homogeneity analysis, ensuring that each dimension adequately represents the theoretical construct. This approach strengthens construct validity and reliability, which are critical for accurate interpretation in research and clinical practice.

In summary, the EFA helps explore potential factor structures in the data and reduce complexity; however, it does not test theory-driven models, which would require confirmatory factor analysis (CFA) [20,21,22,23].

## 3. Results

Table 1 shows the descriptive results of the total Soft Skills Questionnaire and its dimensions (communication skills, confidentiality, and management and emotional intelligence). Means, standard deviation, kurtosis, and skewness for both samples are included in the same table.

### 3.1. Reliability and Exploratory Analysis

Exploratory factor analysis (EFA) was conducted to examine the underlying factor structure of the Soft Skills Questionnaire in sample 1. The Kaiser–Meyer–Olkin (KMO) values ranged from 0.57 to 0.62 and Bartlett’s test of sphericity was significant at X^2^ = 384.46, *p* < 0.001 (*p* < 0.001), indicating that the data were suitable for factor analysis. However, given the relatively low KMO values in some dimensions, these analyses are presented for exploratory purposes and should not be interpreted as definitive evidence of the scale’s structural validity. For the communication skills dimension, results were: KMO = 0.62 and significance for Bartlett’s test of sphericity, X^2^ = 384.46, *p* < 0.001. Cronbach’s alpha (α) value for this dimension was 0.51 and McDonald’s omega was 0.55, indicating, in both cases, that the internal consistency between items is very low. This variance explained why the percentage for communications skills was 0.14. The factorial weight ranged between 0.11 and 0.67.

The confidentiality dimension was composed of six items. KMO value was 0.61 and significance for Bartlett’s test of sphericity, X^2^ = 95.80, *p* < 0.001. The Cronbach’s alpha value for this dimension was 0.48 and McDonald’s omega was 0.43, indicating, in both cases, that the internal consistency between items is very low. The variance explained in the percentage for communications skills was 0.18. The factorial weight ranged between 0.13 and 0.61.

Finally, the third dimension of management and emotional intelligence was also composed of six items. KMO value was 0.57 and significance for Bartlett’s test of sphericity, X^2^ = 85.36, *p* < 0.001. The Cronbach’s alpha value for this dimension was 0.43 and McDonald’s omega was 0.41, indicating, in both cases, that the internal consistency between items is very low. The variance explained in the percentage for communications skills was 0.19. The factorial weight ranged between 0.23 and 0.59.

Regarding the reliability of the whole questionnaire in Sample 1, Cronbach’s alpha was 0.65 and McDonald’s omega was 0.64, indicating more acceptable consistency at the global-scale level. These reliability indices were calculated using only the items belonging to each specific dimension, as required in multidimensional instruments. These findings highlight potential limitations of the instrument and are presented as preliminary, exploratory observations rather than as definitive evidence of its psychometric properties (Table 2).

For sample 1, the correlations factors were the following: communication skills and confidentiality (r=−0.001, p=0.98); communication skills also showed no meaningful relationships with management and emotional intelligence (r=0.04, p=0.50). However, confidentiality correlated moderately and significantly with management and emotional intelligence (r=0.26, p<0.001). The total soft-skills score displayed strong and statistically significant associations with all three dimensions—communication skills (r=0.78, p<0.001), confidentiality (r=0.49, p<0.001), and management and emotional intelligence $\left(r=0.54,\;p<0.001\right)$—indicating substantial shared variance across the components of the scale.

The second sample the communication skills dimension showed a KMO index of 0.67 and significance for Barlett’s test of sphericity, X^2^ = 213.26, *p* < 0.001. The Cronbach’s alpha value was 0.57 and McDonald’s omega was 0.57, indicating, in both cases, that the internal consistency between items is very low. The variance explained in the percentage for communications skills was 0.18. The factorial weight ranged between 0.14 and 0.65.

For the second sample (university students) the confidentiality dimension showed a KMO index of 0.49 and significance for Barlett’s test of sphericity, X^2^ = 38.28, *p* < 0.001. The Cronbach’s alpha value was 0.25 and for McDonald’s omega was 0.35, indicating, in both cases, that the internal consistency between items is very low. The variance explained in the percentage for communications skills was 0.20. The factorial weight ranged between 0.03 and 1.

For the second sample, the management and emotional intelligence dimension showed a KMO index of 0.60 and significance for Barlett’s test of sphericity, X^2^ = 40.91, *p* < 0.001. The Cronbach’s alpha value was 0.44 and McDonald’s omega was 0.48, indicating, in both cases, that the internal consistency between items is inadequate. The variance explained in the percentage for communications skills was 0.18. The factorial weight ranged between 0.08 and 72.

For the whole questionnaire the Cronbach’s alpha value was 0.65 and McDonald’s omega was 0.64 for sample 1 and, for sample 2, Cronbach’s alpha was 0.70 and McDonald’s omega was 0.70.

The factor correlation for the second sample was: communication skills and confidentiality $\left(r=0.002,\;p=0.98\right)$; communication skills also showed no meaningful association with management and emotional intelligence $\left(p>0.05\right)$. On the other hand, confidentiality correlated significantly with management and emotional intelligence $\left(<0.001\right)$. The total soft-skills score again showed strong and statistically significant relationships with all three dimensions—communication skills $\left(r=0.71,\;p<0.001\right)$, confidentiality $\left(r=0.54,\;p<0.001\right)$, and management and emotional intelligence $\left(r=0.68,\;p<0.001\right)$.

### 3.2. Exploratory Associations with Burnout

Table 3 summarizes weak correlations between the soft-skills scale (for the sample of nurses) and the burnout dimensions of emotional exhaustion and depersonalization. Only the management and emotional intelligence subscale showed a small but significant positive correlation with emotional exhaustion (r = 0.18, *p* < 0.05), suggesting a possible link between emotional management and higher emotional strain. The remaining dimensions showed no significant correlation.

These findings are presented as preliminary observations, highlighting possible links between specific soft skills and aspects of professional stress, rather than as evidence of criterion validity.

## 4. Discussion

This study aimed to translate the SSQN into Spanish and critically explore its empirical behavior within the field of professional nursing. The inclusion of two distinct groups—nursing students and practicing nurses—made it possible to explore the instrument’s applicability at different stages of educational and professional development.

The exploratory analyses revealed heterogeneous patterns regarding the questionnaire’s factorial structure and internal consistency. These findings allowed the identification of potentially useful elements but also revealed structural inconsistencies that call for a reconsideration of the instrument’s design and its suitability for use in real clinical settings. The SSQN’s three-dimensional structure does not fully capture the conceptual breadth of soft skills, potentially affecting the observed patterns in internal consistency and factor structure. The inconsistencies observed suggest that revising certain items could enhance the internal coherence of the dimensions and improve the overall precision of the questionnaire [24,25,26,27,28].

In this regard, the findings underscore the need to review and refine certain items and to incorporate methodological strategies that could capture the complexity of soft skills in nursing practice more faithfully. This proposal seeks not only to improve a specific tool but also to open a broader debate about the standards required to rigorously assess these key competencies in professional care. 

Exploratory analyses highlighted that certain dimensions of the SSQN do not fully align with the theoretical breadth of soft skills. The KMO index fell below acceptable thresholds, indicating that the data were not suitable for factor analysis and that both the sample and the instrument’s structure may require reconsideration. Internal consistency was low in the subscales, highlighting areas for refinement, rather than providing a definitive measure of reliability. Rather than functioning as coherent indicators of interpersonal, emotional, or ethical competencies, the items appear to operate inconsistently, preventing the instrument from being considered a reliable measurement tool in its current form. These methodological findings are not merely technical issues; they reflect broader conceptual challenges inherent to the assessment of soft skills and underscore the need to reconsider how these competencies are defined, operationalized, and measured in nursing practice. The partial mismatch between the theoretical scope of soft skills and the three-dimensional structure of the SSQN further reinforces the need for substantial revision of the instrument to achieve better alignment between theoretical expectations and empirical performance [29,30].

Exploratory associations with burnout were examined, but these findings reflect limitations of the instrument rather than substantive relationships with external constructs. These findings are interpreted as preliminary observations and are not evidence of criterion validity. Instead, they reflect the limitations of the instrument itself rather than any substantive relationship with external constructs.

Furthermore, the present findings highlight a partial mismatch between the theoretical scope of social skills, as described in the introduction, and the empirical structure of the SSQN. While social skills in nursing encompass a wide range of interpersonal, emotional, cognitive, and ethical competencies, the three-dimensional model of the SSQN represents only a subset of this conceptual spectrum. Areas that are frequently highlighted in literature, such as teamwork or decision-making, are not explicitly reflected in the instrument, while ‘confidentiality,’ which is essential in professional ethics, appears as a separate dimension. This conceptual imbalance may partly explain the inconsistencies observed in the factor structure and internal consistency indices and reinforces the need to revise the instrument to achieve greater alignment between theoretical expectations and empirical measurement.

With respect to content validity, the data collected provide a useful basis for further development of the instrument. Although the items were translated and reviewed to ensure linguistic adequacy, some of them appeared to fall short of optimal clarity or representativeness. This may have led to variable interpretations influenced by professional context, prior experience, or participants’ educational level.

Therefore, it is advisable to thoroughly review items showing semantic overlap or ambiguous wording to ensure more uniform interpretation. Future studies could incorporate more systematic procedures for content evaluation, such as involving a broader range of clinical, educational, and cultural perspectives, to strengthen the conceptual and practical relevance of the instrument [31].

A relevant aspect to consider is the possible influence of social desirability on the responses, particularly among the professional sample. Since the questionnaire addresses dimensions related to values, attitudes, and competencies inherent to the nursing role, it is understandable that some participants may have responded in line with social expectations or professional ideals. This phenomenon, common in self-report studies, should be understood as an inherent methodological feature rather than solely as a limitation [32,33,34,35]. Nevertheless, for future applications, it is recommended to complement data collection with strategies designed to identify or control this type of bias—such as specific scales or multimethod designs—in order to distinguish more clearly between normative responses and personal perceptions [34,36,37].

Overall, this study provides a foundation for reflecting critically on the assessment of interpersonal, emotional, and ethical competencies and highlights the importance of refining the SSQN before wider use in Spanish-speaking populations.

### Limitations

This study has several limitations that should be considered when interpreting the results.

First, the sample was obtained from a single hospital using non-probability convenience sampling. The organizational and training characteristics of the center may have influenced the interpretation of the items, limiting the generalizability of the findings to other institutional contexts.

Second, there is a relevant conceptual limitation. Although the introduction describes the theoretical breadth of the soft skills construct, this complexity is not fully reflected in the three-dimensional structure of the SSQN. This discrepancy between the construct and the instrument may have affected the factor structure and internal consistency indices observed.

There was also an imbalance in the size of the participating groups, with considerably fewer students than professionals. Although this difference reflects the actual availability of the population and is justified in the methodology, it remains a limitation because it reduces the comparability between groups and may affect the statistical robustness of some analyses.

In addition, the exclusively quantitative approach prevented an in-depth exploration of the perceptions and meanings that participants attribute to the items, which is particularly relevant given the lack of consensus on what competencies constitute soft skills and how they should be assessed.

Another methodological limitation relates to the presence of the researcher during the first phase of data collection (up to 50% of the sample). Although this intervention facilitated participation and allowed questions to be answered, it could also have generated social desirability bias or influenced the spontaneity of responses. Future research should standardize the conditions of administration and minimize external intervention.

Finally, some statistical assumptions were not adequately met, such as the KMO values for the application of exploratory factor analysis. Nevertheless, the analysis was carried out, revealing that certain dimensions of the instrument require revision. Some items showed low relevance and weak correlations and should therefore be eliminated or reformulated in future studies. These limitations may also have contributed to the reduced correlations with the criterion instrument used.

Overall, the results suggest that quantitative instruments alone do not fully capture the complexity of soft skills. It would be advisable to incorporate qualitative methods and observation techniques that allow for the collection of contextual, emotional, and relational aspects that escape structured measures. Furthermore, the lack of theoretical consensus on the definition and assessment of soft skills underscores the need for more integrative approaches that are sensitive to cultural and professional variations [38,39].

Recommendations for future research:

Refine or eliminate items with low factor loadings.

Conduct multicenter studies to increase the representativeness of the sample.

Complement the evaluation with qualitative methods that capture cultural and contextual nuances.

Balance group sizes to improve comparability.

Reduce researcher intervention during data collection to encourage more authentic responses.

## 5. Conclusions

Soft skills are widely recognized as essential for ensuring high-quality and humanized nursing care, particularly in healthcare contexts increasingly shaped by technological demands. However, their assessment remains challenging due to their subjective, multidimensional, and context-dependent nature. In this regard, the present study offers the first Spanish translation of the SSQN and provides a critical exploratory examination of its psychometric performance, generating an initial framework for reflecting on the evaluation of interpersonal, emotional, and ethical competencies in both nursing students and professionals.

The results provide valuable information about the structure and reliability of the instrument, while revealing several areas that require substantial improvement. Observed low reliability indices, variable KMO values, and inconsistencies in factor structure highlight areas for refinement rather than providing definitive evidence regarding the instrument’s measurement properties. Consequently, the findings should be interpreted cautiously, with the factor analyses considered exploratory and indicative rather than confirmatory. The partial mismatch between the theoretical concept of social skills and the three-dimensional structure of the SSQN suggests the need for further conceptual clarification and item revision. Similarly, methodological limitations restrict the generalizability of the results, but offer clear guidance for future research directions.

Overall, the study highlights the need to move toward more comprehensive assessment strategies that integrate quantitative and qualitative approaches and more accurately capture the interpersonal, emotional, and ethical nuances of social skills in nursing practice. Strengthening these assessment frameworks will be essential to supporting meaningful training, enhancing professional development, and promoting truly person-centered care.

## Figures and Tables

**Table 1:** Descriptive results of soft-skills scale and its dimensions.

			Sample 1					Sample 2		
	M		SD	Kurtosis	Skewness	M		SD	Kurtosis	Skewness
Whole questionnaire	83.49	3.87	6.42	4.04	0.80	84.25	3.91	6.43	4.90	1.31
Communications skills	50.28	2.81	4.87	1.85	−0.55	50.83	2.41	4.16	1.12	−0.34
Confidentiality	16.83	2.73	2.47	7.42	1.65	14.47	3.16	2.58	0.89	0.43
Management and emotional intelligence	16.38	3.34	2.62	1.53	0.22	18.96	3.37	3.06	2.98	0.85

**Table 2:** Reliability and homogeneity indices of the Soft Skills Questionnaire and its dimensions.

	Sample 1			Sample 2		
	ω	α	Range of HI	ω	α	Range of HI *
Whole questionnaire	0.64	0.65	0.09–0.48	0.70	0.70	0.08–0.42
Communications skills	0.51	0.55	0.05–0.44	0.57	0.57	0.07–0.46
Confidentiality	0.48	0.43	0.08–0.39	0.35	0.25	0.04–0.21
Management and emotional intelligence	0.43	0.41	0.02–0.32	0.48	0.44	0.08–0.39

* HI: homogeneity indices.

**Table 3:** Correlations of the soft-skills scale, its dimensions, and the dimensions of Maslach’s scale.

	Emotional Exhaustion	SE	Depersonalization	SE
Whole questionnaire	0.05	0.0632	0.03	0.0632
Communications skills	0.05	0.0632	0.07	0.0631
Confidentiality	0.09	0.0629	0.06	0.0621
Management and emotional intelligence	0.18 *	0.0620	−0.03	0.0632

* *p* < 0.05; SE = standard error.

## Data Availability

The data presented in this study are available on request from the corresponding author. The data are not publicly available due to privacy restrictions.
